# Polybrominated Diphenyl Ethers (PBDEs) in PM_1_ of Residential Indoor Air: Levels, Seasonal Variability, and Inhalation Exposure Assessment

**DOI:** 10.3390/jox15060195

**Published:** 2025-11-12

**Authors:** Darija Klinčić, Karla Jagić Nemčić, Ivana Jakovljević, Marija Jelena Lovrić Štefiček, Marija Dvoršćak

**Affiliations:** Division of Environmental Hygiene, Institute for Medical Research and Occupational Health, Ksaverska Cesta 2, HR-10000 Zagreb, Croatia; dklincic@imi.hr (D.K.); kjagic@imi.hr (K.J.N.); ijakovljevic@imi.hr (I.J.); mlovric@imi.hr (M.J.L.Š.)

**Keywords:** polybrominated diphenyl ethers (PBDEs), fine particulate matter PM_1_, indoor air, seasonal variation, human exposure assessment, residential environments

## Abstract

Indoor exposure to polybrominated diphenyl ethers (PBDEs), particularly those bound to fine particulate matter (PM_1_, particles < 1 µm), may pose a health concern, especially in light of prolonged indoor occupancy and the capacity of ultrafine particles to reach the lower respiratory tract. This study investigates indoor exposure to PBDEs associated with PM_1_ in residential homes in Zagreb, Croatia, across warm and cold seasons. BDE-47 was consistently detected in all samples, while BDE-183 was consistently absent. Elevated concentrations and increased detection frequencies of BDE-99 and BDE-100 were observed during the colder season. Consequently, total PBDE (ΣPBDE) levels in the cold season were approximately 2.5 times higher than in the warm season. Although estimated daily inhalation intakes were below established oral reference doses, the potential for deep pulmonary deposition and systemic distribution underscores the need for further investigation. These findings represent the first reported data on indoor PM_1_-associated PBDEs in Europe, emphasizing the impact of seasonal dynamics on inhalation exposure due to variation on indoor contaminant levels.

## 1. Introduction

Polybrominated diphenyl ethers (PBDEs) are a group of synthetic organobromine compounds that have been widely used as additives in commercial products to enhance their fire resistance, thereby reducing human casualties and material losses caused by fires. Their release from products throughout their lifecycle, from manufacturing to disposal, has led to their ubiquitous presence in environment, and although the production and use of PBDEs were phased out approximately twenty years ago (depending on the specific commercial mixture), these compounds are still detected in various environmental matrices, including indoor air and dust [1]. Due to their physicochemical characteristics and persistence, they have been proven to be harmful to human health, acting as endocrine disruptors and being linked to neurodevelopmental, reproductive, and carcinogenic effects.

Dietary intake and ingestion of indoor dust represent the primary pathways of human exposure to PBDEs, while inhalation and dermal absorption contribute to a lesser extent. However, the inhalation of fine particles capable of penetrating deeply into the respiratory system poses a significant health risk [2,3]. Due to their higher surface area-to-volume ratio, smaller particles have greater potential for adverse biological interactions with respiratory tissues [2]. It has been demonstrated that PBDEs are predominantly associated with smaller airborne particles [4,5,6], emphasizing the importance of studying PBDE concentrations related to smaller fractions of particulate matter (PM), especially those with an equivalent aerodynamic diameter less than 2.5 µm (PM_2.5_) or even less than 1 µm (PM_1_). While some data on PBDE concentrations bound to particulate matter of different sizes in ambient air [5,7,8,9,10,11,12], or in occupational indoor environments [4,6] exist, corresponding data from residential indoor environments remain scarce. Richman et al. [5] reported that in size-fractionated PM samples from three Canadian homes, more than 50% of the PBDE mass was associated with particles smaller than 1 μm, consistent with the enrichment of PBDEs in fine outdoor air particles [8,10,11]. PM_1_ measurements of associated PBDEs are important since PM_1_ have higher deposition rates in the respiratory tract and lungs compared to larger airborne particulate matter, highlighting potential endocrine, neurodevelopmental, and liver-related health effects from inhalation that may differ from ingestion [4,5]. Regulatory frameworks mainly focus on PM_10_ and PM_2_._5_ fractions; however, scientific data highlights how important it is to consider the chemical content of smaller particles like PM_1_ [13]. Focusing on the PM_1_ fraction when assessing health concerns is further supported by toxicological studies showing it is more harmful than PM_2_._5_ in terms of cytotoxic effects and inflammation [14,15].

In general, indoor air quality has become a critical public health concern, as individuals typically spend between 80% and 90% of their time indoors, with the majority of that time in residential environments [16,17]. Indoor air can contain various pollutants originating from building materials and consumer products, but also different occupant activities, with concentrations exceeding those found outdoors [18,19]. Indoor air quality has been associated with a range of adverse health outcomes, including respiratory and cardiovascular diseases, allergies, neurodevelopmental disorders, and cancer [17].

Obtaining data on indoor contaminant levels is crucial given the substantial proportion of time spent indoors and the potential for elevated pollutant levels. To the best of our knowledge, this study represents the first European investigation of PBDE concentrations in PM_1_ in indoor residential settings, and the first to encompass multiple homes across both, cold and warm, seasons. Additionally, the collected data were utilized to assess the potential health risks for adults and toddlers exposed via inhalation which can serve for development of effective risk reduction and regulatory strategies.

## 2. Materials and Methods

### 2.1. Samples

Samples for PBDE analysis were collected as part of the Zagreb pilot study within the EDIAQI (Evidence Driven Indoor Air Quality Improvement) project [15,20]. A low-volume sampler (MiniVol Portable Air Sampler, AirMetrics, Eugene, OR, USA) was used to collect the PM_1_ fraction of particulate matter onto quartz fiber filters (47 mm diameter, Whatman^®^ QM-A, Tisch Scientific, Cleves, OH, USA) at a flow rate of about 5 L min^−1^ continuously over seven consecutive days. The samplers were placed in the middle of the living room, as far away from the windows and kitchen entrance as possible. The household members were instructed to open their windows in accordance with their usual habits. After collection, the filters were stored at −20 °C until analysis. Half of the filter was used for the analysis of PBDE compounds.

Particulate matter sampling was performed in 15 residential homes in Zagreb, Croatia, during the colder season (November 2023–February 2024, average monthly outdoor temperature ranged between 1.6 °C and 8.8 °C), and repeated during the warmer season (July and August 2023 and 2024, average monthly outdoor temperature ranged between 23.1 °C and 24.6 °C), resulting in a total of 30 filters collected.

### 2.2. Chemical Analysis

Prior to extraction, the filters were spiked with surrogate standards (BDE-77 and BDE-128). Each sample was extracted with 15 mL of an *n*-hexane:acetone solvent mixture (1:1, *v*/*v*) by ultrasonication for 20 min. The supernatant was separated, and extraction was repeated with an additional 10 mL of the same solvent mixture for 10 min. The combined extracts were concentrated under a gentle nitrogen flow to approximately 2 mL.

The extract was then cleaned by mixing with concentrated sulfuric acid, evaporated to incipient dryness under nitrogen, and reconstituted in 200 µL of *n*-hexane for subsequent analysis.

Seven PBDE congeners (BDE-28, -47, -99, -100, -153, -154, and -183) were quantified by tandem mass spectrometry (GC-MS/MS) using an Agilent 7010B system coupled to an Agilent 7890B gas chromatograph. GC-MS/MS was operated in electron ionization mode with multiple reaction monitoring (MRM). Chromatographic separation was performed using an Agilent DB-5ms capillary column (15 m length, 250 μm internal diameter, 0.25 μm film thickness).

The average analytical recovery of the employed method was 77%, with the RSD values below 4% indicating good method precision. The limit of detection (LOD) for the overall method, defined as a signal-to-noise ratio of 3 (S/N = 3), was estimated employing real samples, and the obtained value was 0.005 pg m^−3^.

## 3. Results

### 3.1. Indoor Air Concentrations of PBDEs Associated with PM_1_ and Seasonal Variations

All analyzed samples contained detectable levels of at least one targeted PBDE congener. Among the PBDE congeners analyzed, BDE-47 was the only compound detected in all samples, while BDE-183 was not detected in any sample (Table 1). The detection frequencies of BDE-99 and BDE-100 showed notable seasonal variation, with higher detection rates observed during the colder season. In the warmer period, BDE-47 exhibited the highest median concentration, being the only congener detected in over 50% of samples. Conversely, during the colder season, BDE-99 showed the highest mass concentration and median concentration, surpassing BDE-47.

**Figure 1:**
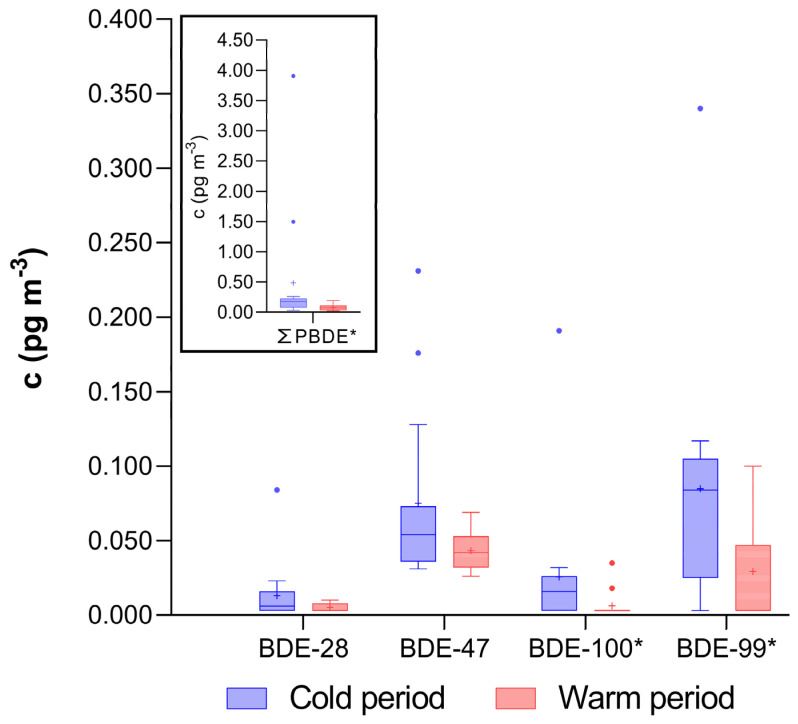
Concentration of individual PBDE congeners (excluding BDE-153, BDE-154, and BDE-183, as their concentrations were below the LOD in over 80% of the samples) and sum (ΣPBDE) in PM_1_ fraction of indoor air obtained in 15 homes from Zagreb, Croatia. The data are presented as median (line), mean (+), 25th and 75th percentile (box), and interquartile range (whisker), and outliers (•). * Significantly higher concentrations in colder period (Wilcoxon Matched Pairs Test; *p* < 0.05).

The total ΣPBDE concentration during the colder period was approximately 2.5 times higher than during the warmer period (Figure 1), a difference that was statistically significant (*p* < 0.05; Wilcoxon Matched Pairs Test). The seasonal difference was primarily driven by BDE-99 and BDE-100, as the concentrations and detection frequencies of the other congeners remained relatively stable across seasons.

### 3.2. Estimated Daily Intake (EDI) via Inhalation and Risk Characterization

The estimated daily intake (EDI) via inhalation was calculated using the following equation:

$${EDI}_{inhalation}=\frac{c\times IR\times HEF}{BW}[pg\;{kg}^{-1}\;{day}^{-1}]$$

where

- *c* is the median PBDE PM_1_ concentration (pg m^−3^);
- *IR* is the daily inhalation rate (8 m^3^ day^−1^ for toddlers and 16 m^3^ day^−1^ for adults) [1,21];
- *HEF* is the home exposure fraction (86% for toddlers and 64% for adults) [22];
- *BW* is the body weight (13.8 kg for toddlers and 80 kg for adults) [21].

Assuming 100% absorption, the calculated EDIs were:

- Warmer period: 0.035 pg kg^−1^ day^−1^ (toddlers), 0.009 pg kg^−1^ day^−1^ (adults);
- Colder period: 0.088 pg kg^−1^ day^−1^ (toddlers), 0.023 pg kg^−1^ day^−1^ (adults).

## 4. Discussion

Indoor air quality has become an increasing focus of environmental health research, particularly as the majority of people’s time is now spent indoors. Fine particulate matter, capable of depositing deep within the respiratory system and transporting organic pollutants, poses a particular concern for human health. Despite its importance, prior data on PBDE concentrations bound to PM_1_ in residential indoor air were lacking.

The consistent detection of BDE-47 across all samples indicates its widespread presence in indoor environments, aligning with its prevalence in the pentaBDE commercial formulation. Additionally, dominance of BDE-99, another component of pentaBDE commercial mixture, in the colder period, points to common indoor sources such as upholstered furniture, textiles, and electronics. On the other hand, absence of BDE-183—despite its previous detection in settled dust from Zagreb homes [23,24]—suggests differential partitioning behavior between airborne PM_1_ and settled dust, likely influenced by compound-specific physicochemical properties. Accordingly, the average concentration of BDE-183 in the total indoor airborne particulate matter in households in Izmir, Turkey, was below the method detection limit, while it was measurable in matching house dust samples [25]. Such a result is consistent with the findings of de la Torre et al. [8], who obtained that PBDE congener contribution increased with both particle size and bromination degree.

The detection frequencies of BDE-99 and BDE-100 showed notable seasonal variation, with higher detection rates observed during the colder season. During the warmer period, BDE-47 exhibited the highest median concentration, attributable to it being the only congener detected in more than 50% of samples. In contrast, during the colder season, BDE-99 not only showed the highest mass concentration in individual samples but also surpassed BDE-47 in terms of median concentration. These findings highlight the potential influence of indoor activities, and primarily of ventilation habits of occupants on PBDE levels associated with airborne particulate matter.

To our knowledge, this is the first European study to report PBDE concentrations associated with the PM_1_ fraction of indoor residential air. A similar study by Richman et al. [5] examined only three Canadian homes, detecting BDE-47, -99, -100, and -183, with total concentrations across all particle sizes ranging from 8.7 to 15.7 pg m^−3^. Accounting for their observation that over 50% of PBDE mass was associated with particles smaller than 1 μm, their values remain higher than those reported here.

Direct comparison with other available literature, however, remains challenging due to differences in sampling strategies, particle size cut-offs, analytical methods, and the congeners included in the analysis. As we mentioned before, PBDEs are primarily associated with smaller airborne particles. Also, there are suggestions that PM_1_ accounts for a substantial proportion of the total mass of larger particle fractions, particularly PM_2.5_ [3,26]. However, to the best of our knowledge, and based on currently available literature, no studies have reported PBDE concentrations in PM_1_, or even PM_2.5_ sampled in residential indoor environments in Europe. There is a study reporting PBDE levels in PM_1_ and PM_10_ fractions sampled in the same computer repair service [4]. Considering the type of indoor area in which the samples were collected, the reported PBDE concentrations are several times higher than in our study. An interesting result of this study was that PBDE concentrations in PM_10_ were only about twice those in PM_1_, despite a nine-fold difference in particle mass, confirming that submicron particles represent a significant pathway for particle-phase PBDE exposure [4]. There are few more studies investigating PBDE concentrations in PM_1_ or PM_2.5_ fractions sampled in various indoor environments [19,27,28,29], but none of them included households. Due to the different purpose of such environments, different potential sources of PBDEs, and differences in the time people spend in these spaces, we believe that comparison of these results with our households’ data would not be scientifically justified.

Since specific inhalation reference doses (RfD) for PBDEs are unavailable, the oral RfD for pentaBDE (2000 ng kg^−1^ day^−1^) [30] was used for a preliminary risk assessment. The obtained EDIs for ΣPBDEs were several orders of magnitude lower than the oral RfD, suggesting a negligible health risk via inhalation. However, this risk characterization must be interpreted with caution. Inhaled particles, particularly those within the PM_1_ range, bypass hepatic metabolism and directly enter systemic circulation through the pulmonary alveoli. Therefore, potential toxicological effects may differ from those predicted based solely on oral exposure assessments. Furthermore, personal exposure levels are often significantly higher than those measured by stationary indoor monitors, due to human activities that resuspend settled dust [31].

The seasonal shift in the contribution of individual congeners to total intake further reflects the measured concentration trends. In the warmer period, BDE-47 contributed approximately 60% to the total EDI, while during the colder period, its contribution decreased to about 30%, with BDE-99 emerging as the dominant contributor at 48%. These results underline the importance of considering seasonal variability and individual congener profiles when assessing indoor exposure to PBDEs.

## 5. Conclusions

This study provides the first data on polybrominated diphenyl ethers associated with the PM_1_ fraction in residential indoor air in Europe. Our results demonstrate that indoor PBDE concentrations are higher during the colder season, likely due to reduced ventilation and increased use of potential sources like electronic devices. Although the estimated daily intakes (EDIs) via inhalation were several orders of magnitude lower than the established oral reference dose, the potential for deeper respiratory deposition and systemic exposure warrants the need for continued research. Seasonal variations, and the contribution of individual congeners to overall exposure underscore the necessity of comprehensive indoor air assessments. Our future studies will involve a larger set of indoor air samples alongside simultaneous outdoor measurements to better characterize indoor–outdoor relationships, seasonal variations and source contributions of PBDEs.

## Figures and Tables

**Table 1:** Mass concentrations of PBDEs (pg m^−3^) in PM_1_ sampled in warmer and colder period in same residential indoor spaces in Zagreb, Croatia.

	Warmer Period (*N* = 15)		Colder Period (*N* = 15)	
Compound	Detection Frequency/%	Range (Median *)	Detection Frequency/%	Range (Median *)
BDE-28	40	<LOD–0.010	53	<LOD–0.084 (0.006)
BDE-47	100	0.026–0.069 (0.042)	100	0.031–0.231 (0.054)
BDE-99	47	<LOD–0.100	80	<LOD–0.340 (0.084)
BDE-100	13	<LOD–0.035	67	<LOD–0.191 (0.016)
BDE-153	0		13	<LOD–3.171
BDE-154	0		20	<LOD–0.357
BDE-183	0		0	
ΣPBDEs	100	0.026–0.193 (0.071)	100	0.033–3.904 (0.177)

*N* = number of samples; * Median value is shown only for compounds detected in ≥50% of analyzed samples.

## Data Availability

The original contributions presented in this study are included in the article. Further inquiries can be directed to the corresponding author(s).

## References

[1] Besis A., Samara C. (2012). Polybrominated Diphenyl Ethers (PBDEs) in the Indoor and Outdoor Environments—A Review on Occurrence and Human Exposure. Environ. Pollut..

[2] Mei M., Song H., Chen L., Hu B., Bai R., Xu D., Liu Y., Zhao Y., Chen C. (2018). Early-Life Exposure to Three Size-Fractionated Ultrafine and Fine Atmospheric Particulates in Beijing Exacerbates Asthma Development in Mature Mice. Part. Fibre Toxicol..

[3] Yang M., Guo Y.M., Bloom M.S., Dharmagee S.C., Morawska L., Heinrich J., Jalaludin B., Markevychd I., Knibbsf L.D., Lin S. (2020). Is PM1 Similar to PM2.5? A New Insight into the Association of PM1 and PM2.5 with Children’s Lung Function. Environ. Int..

[4] Genisoglu M., Sofuoglu A., Kurt-Karakus P.B., Birgul A., Sofuoglu S.C. (2019). Brominated Flame Retardants in a Computer Technical Service: Indoor Air Gas Phase, Submicron (PM1) and Coarse (PM10) Particles, Associated Inhalation Exposure, and Settled Dust. Chemosphere.

[5] Richman K.E., Butt C.M., Young C.J. (2018). Size-Resolved Particle Measurements of Polybrominated Diphenyl Ethers Indoors: Implications for Sources and Human Exposure. Environ. Toxicol. Chem..

[6] Jin M., Guo J., Xu Z., Sun L., Zhang S. (2025). Pollution Status, Phase Partitioning, Potential Sources, and Health Impacts of Polybrominated Diphenyl Ethers in Hangzhou Offices. Atmos. Environ..

[7] Besis A., Botsaropoulou E., Voutsa D., Samara C. (2015). Particle-Size Distribution of Polybrominated Diphenyl Ethers (PBDEs) in the Urban Agglomeration of Thessaloniki, Northern Greece. Atmos. Environ..

[8] de la Torre A., Barbas B., Sanz P., Navarro I., Artíñano B., Martínez M.A. (2018). Traditional and Novel Halogenated Flame Retardants in Urban Ambient Air: Gas-Particle Partitioning, Size Distribution and Health Implications. Sci. Total Environ..

[9] Li Z., Zhu Y., Wang D., Zhang X., Jones K.C., Ma J., Wang P., Yang R., Li Y., Pei Z. (2021). Modeling of Flame Retardants in Typical Urban Indoor Environments in China during 2010-2030: Influence of Policy and Decoration and Implications for Human Exposure. Environ. Sci. Technol..

[10] Mandalakis M., Besis A., Stephanou E.G. (2009). Particle-Size Distribution and Gas/Particle Partitioning of Atmospheric Polybrominated Diphenyl Ethers in Urban Areas of Greece. Environ. Pollut..

[11] Okonski K., Degrendele C., Melymuk L., Landlová L., Kukučka P., Vojta Š., Kohoutek J., Čupr P., Klánová J. (2014). Particle Size Distribution of Halogenated Flame Retardants and Implications for Atmospheric Deposition and Transport. Environ. Sci. Technol..

[12] Zhang Y., Wu M., Xu M., Hu P., Xu X., Liu X., Cai W., Xia J., Wu D., Xu X. (2022). Distribution of Flame Retardants among Indoor Dust, Airborne Particles and Vapour Phase from Beijing: Spatial–Temporal Variation and Human Exposure Characteristics. Environ. Int..

[13] Squizzato S., Masiol M., Agostini C., Visin F., Formenton G., Harrison R.M., Rampazzo G. (2016). Factors, Origin and Sources Affecting PM1 Concentrations and Composition at an Urban Background Site. Atmos. Res..

[14] Jalava P.I., Happo M.S., Huttunen K., Sillanpää M., Hillamo R., Salonen R.O., Hirvonen M.R. (2015). Chemical and Microbial Components of Urban Air PM Cause Seasonal Variation of Toxicological Activity. Environ. Toxicol. Pharmacol..

[15] Lovrić M., Gajski G., Fernandez-Agüera J., Pöhlker M., Gurcsh H., Borg A., Switters J., Mureddu F., The EDIAQI Consortium (2025). Evidence Driven Indoor Air Quality Improvement: An Innovative and Interdisciplinary Approach to Improving Indoor Air Quality. Biofactors.

[16] Klepeis N.E., Nelson W.C., Ott W.R., Robinson J.P., Tsang A.M., Switzer P., Behar J.V., Hern S.C., Engelmann W.H. (2001). The National Human Activity Pattern Survey (NHAPS): A Resource for Assessing Exposure to Environmental Pollutants. J. Expo. Anal. Environ. Epidemiol..

[17] WHO (2010). WHO Guidelines for Indoor Air Quality: Selected Pollutants.

[18] Weschler C.J., Nazaroff W.W. (2008). Semivolatile Organic Compounds in Indoor Environments. Atmos. Environ..

[19] Chao H.R., Que D.E., Gou Y.Y., Chuang C.Y., Chang T.Y., Hsu Y.C. (2016). Indoor and Outdoor Concentrations of Polybrominated Diphenyl Ethers on Respirable Particulate in Central and Southern Taiwan. Aerosol Air Qual. Res..

[20] Lovrić M., Račić N., Pehnec G., Jakovljević I. (2024). Indoor Polycyclic Aromatic Hydrocarbons—Relationship to Ambient Air, Risk Estimation, and Source Apportionment Based on Household Measurements. Atmosphere.

[21] US EPA (2011). 2011 Exposure Factors Handbook: 2011 Edition.

[22] Pawar G., Abdallah M.A.E., De Sáa E.V., Harrad S. (2017). Dermal Bioaccessibility of Flame Retardants from Indoor Dust and the Influence of Topically Applied Cosmetics. J. Expo. Sci. Environ. Epidemiol..

[23] Jagić K., Dvoršćak M., Tariba Lovaković B., Klinčić D. (2024). Polybrominated Diphenyl Ethers in Paired Dust-Breast Milk Samples: Levels, Predictors of Contamination, and Health Risk Assessment for Infants and Mothers. Environ. Toxicol. Pharmacol..

[24] Klinčić D., Tariba Lovaković B., Jagić K., Dvoršćak M. (2021). Polybrominated Diphenyl Ethers and the Multi-Element Profile of House Dust in Croatia: Indoor Sources, Influencing Factors of Their Accumulation and Health Risk Assessment for Humans. Sci. Total Environ..

[25] Genisoglu M., Edebali O., Sofuoglu A., Turgut C., Sofuoglu S.C. (2025). Airborne and Dust-Bound PBDEs Indoors and Outdoors in Izmir, Türkiye: A Multi-Route Exposure—Risk Assessment. Environ. Pollut..

[26] Oliveira M., Slezakova K., Delerue-Matos C., Pereira M.C., Morais S. (2015). Polycyclic Aromatic Hydrocarbons: Levels and Phase Distributions in Preschool Microenvironment. Indoor Air.

[27] Li Y., Chen L., Ngoc D.M., Duan Y.P., Lu Z.B., Wen Z.H., Meng X.Z. (2015). Polybrominated Diphenyl Ethers (PBDEs) in PM2.5, PM10, TSP and Gas Phase in Office Environment in Shanghai, China: Occurrence and Human Exposure. PLoS ONE.

[28] Deng W.J., Zheng H.L., Tsui A.K.Y., Chen X.W. (2016). Measurement and Health Risk Assessment of PM2.5, Flame Retardants, Carbonyls and Black Carbon in Indoor and Outdoor Air in Kindergartens in Hong Kong. Environ. Int..

[29] Guo J., Lin K., Deng J., Fu X., Xu Z. (2015). Polybrominated Diphenyl Ethers in Indoor Air during Waste TV Recycling Process. J. Hazard. Mater..

[30] https://Iris.Epa.Gov/AdvancedSearch/?Keyword=pbde.

[31] Ferro A.R., Kopperud R.J., Hildemann L.M. (2004). Elevated Personal Exposure to Particulate Matter from Human Activities in a Residence. J. Expo. Anal. Environ. Epidemiol..

